# Reinforcement of Cement Nanocomposites through Optimization of Mixing Ratio between Carbon Nanotube and Polymer Dispersing Agent

**DOI:** 10.3390/polym16030428

**Published:** 2024-02-03

**Authors:** Seok Hwan An, Ki Yun Kim, Jea Uk Lee

**Affiliations:** Department of Advanced Materials Engineering for Information and Electronics, Integrated Education Institute for Frontier Science and Technology (BK21 Four), Kyung Hee University, 1732 Deogyeong-daero, Giheung-gu, Yongin-si 17104, Republic of Korea; ansh0703@khu.ac.kr (S.H.A.); 12rldbs@khu.ac.kr (K.Y.K.)

**Keywords:** carbon nanotube, polymer dispersing agents, centrifugation, cement nanocomposites, compressive strength

## Abstract

Carbon nanotubes (CNTs), known for their exceptional mechanical, thermal, and electrical properties, are being explored as cement nanofillers in the construction field. However, due to the limited water dispersion of CNTs, polymer dispersing agents like polycarboxylate ether (PCE) and sulfonated naphthalene formaldehyde (SNF) are essential for uniform dispersion. In a previous study, PCE and SNF, common cement superplasticizers, effectively dispersed CNTs in cement nanocomposites. However, uncertainties remained regarding the extent to which all dispersing agents interacted efficiently with CNTs. Therefore, this research quantitatively assessed CNT interaction with dispersing agents through dispersion and centrifugation. Approximately 37% of PCE and 50% of SNF persisted compared to CNT after centrifugation. The resulting cement nanocomposites, with optimized mixing ratios, exhibited enhanced compressive strength of about 14% for CNT/PCE (78.13 MPa) and 12.3% for CNT/SNF (76.97 MPa) compared to plain cement (68.52 MPa). XRD results linked strength reinforcement to increased cement hydrate from optimized CNT dispersion. FE-SEM analysis revealed that CNTs were positioned within the pores of the cement. These optimized cement nanocomposites hold promise for improved safety in the construction industry.

## 1. Introduction

Carbon nanotubes (CNTs) exhibit exceptional characteristics, including an extensive specific surface area, excellent electrical and thermal conductivity, as well as remarkable mechanical properties [1,2]. Research on utilizing CNT as a construction material continues, capitalizing on its beneficial properties [3]. CNTs can act as a filler, effectively bridging the gaps among numerous pores formed during cement hydration [4,5]. In addition, owing to their substantial specific surface area, CNTs actively accelerate cement hydration, thereby contributing to the overall enhancement of the mechanical properties of cement [6]. However, CNTs are hydrophobic substances, making them less likely to disperse effectively in aqueous media [7]. To effectively integrate CNTs into cement, overcoming the obstacle of achieving proficient aqueous dispersion is crucial. Kim et al. conducted a study on the enhancement of strength of composite materials, focusing on CNT dispersion methods, and Zou et al. examined changes in interaction between cement and CNT associated with varying sonicator intensity levels [8,9].

In a previous study, to apply CNT to cement, polycarboxylate ether (PCE) and sulfonated naphthalene formaldehyde (SNF), which are polymers used as cement superplasticizers, were employed as polymer dispersing agents to disperse CNT [10]. Contact angle measurements provided evidence that the application of superplasticizers facilitated a closer interaction between CNTs and cement despite their initial incompatibility, thereby demonstrating the effectiveness of this approach. Furthermore, the application of CNT dispersion with dispersing agents demonstrated enhanced strength of the cement nanocomposites compared to plain cement.

To determine the most suitable ratio of a polymer dispersing agent for CNTs, a previous study conducted experiments by fixing the CNT-to-dispersing agent ratio at 1:1, 1:2, and 1:4 [10]. The experiment yielded the most robust results at 1:2, while relatively weak results were obtained at 1:4. The excessive addition of a polymer dispersing agent led to a slowdown in the hydration process of the cement [11]. Based on the above findings, it was suspected that none of the polymer dispersing agents added to the experiment interacted with CNTs. In addition, it was necessary to find the optimal mixing ratio between CNTs and each polymer dispersing agent to improve the mechanical properties of cement nanocomposites.

**Figure 1:**
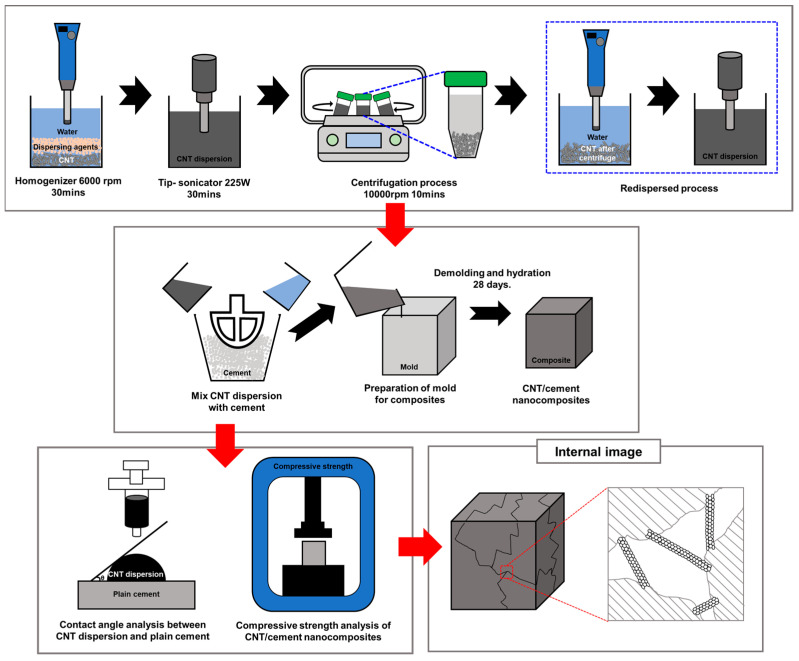
Schematic representation of the entire experimental procedure.

In this study, the compressive strength of the cement nanocomposites, which is the most crucial factor in developing construction materials, has been enhanced by optimizing the mixing ratio between CNTs and polymer dispersing agents (PCE and SNF). For this purpose, dispersion and centrifugation processes were utilized to separate the polymer-dispersing agents that did not interact with CNTs [12]. Additionally, thermogravimetric analysis (TGA) was conducted to determine the ratio of polymer dispersing agents that effectively interacted with CNTs after the centrifugation process. After centrifugation, the CNTs with the polymer dispersing agents attached to their surfaces were subsequently re-dispersed. The degree of dispersion of the CNTs with the polymer dispersing agents was analyzed using optical microscopy (OM) and particle size analysis. Furthermore, field emission transmission electron microscopy (FE-TEM) analysis was used to confirm the distribution of the polymer dispersing agents around the CNTs and their adherence to the CNT surfaces. Contact angle analysis was performed to verify the interfacial affinity between the CNT dispersion and cement, and the mechanical property was measured by fabricating cement nanocomposites and conducting a compressive strength test. X-ray diffraction (XRD) and field emission scanning electron microscopy (FE-SEM) were employed to investigate the internal structure of the cement nanocomposite. Figure 1 displays a schematic overview of the entire experimental process.

## 2. Experimental Section

### 2.1. Materials

Type 1 ordinary Portland cement (Asia Cement, Co., Ltd., Seoul, Republic of Korea) was utilized in conducting the experiment to prepare cement paste with CNTs. Multi-walled carbon nanotubes (MWCNTs, Jenotube 10A, Jeio, Co., Ltd., Incheon, Republic of Korea) were employed, featuring an average diameter of 8–13 nm and an average length of 50–150 µm. For CNTs dispersion in aqueous media, PCE (WD500, KG Chemical, Co., Ltd., Ulju, Republic of Korea) and SNF (Flowmix1, Dong-nam, Co., Ltd., Seongnam, Republic of Korea) served as dispersing agents.

### 2.2. Preparation of CNTs Dispersion and Optimization of Dispersing Agents Content

To assist the dispersion of CNTs, the liquid dispersing agents PCE and SNF were converted into a powdered form using conventional oven drying. Afterward, CNTs and dispersing agents were combined in a consistent weight ratio of 1:2. CNTs and dispersing agents were first placed into deionized water, followed by 30 min treatment at 6000 rpm using a homogenizer (T18 digital ultra turrax, IKA Works, Inc., Staufen, Germany).

Subsequently, the blend was further processed for 30 min with a tip sonicator (ULH 700S, ULSSO Hi-Tech, Co., Ltd., Cheongju, Republic of Korea). The CNT dispersion was subjected to ultrasonic treatment with 225 W of energy, involving a repetitive pattern of 5 s of energy application followed by 5 s of pause while the sonicator tip remained submerged. To optimize the dispersing agent content, the dispersion was treated in a centrifuge (1248, Labogene, Lillerød, Denmark) at 10,000 rpm for 10 min. The sample obtained after a single round of dispersion and centrifugation was designated as CNT/dispersing agent C1, while the sample subjected to two repetitions was labeled as CNT/dispersing agent C2. The CNT/dispersing agent hybrid samples were analyzed by TGA (SDT Q600, TA Instruments, New Castle, DE, USA) to measure the amount of the remaining dispersing agents. Approximately 10 mg of a sample underwent heating at a consistent rate of 10 °C per minute, ranging from 30 to 900 °C, within a nitrogen flow of 100 mL per minute.

### 2.3. Re-Dispersion and Dispersibility Evaluation

A CNT/dispersing agent hybrid dispersion containing 1.4 g of CNTs was prepared by using the CNT/dispersing agent ratio derived from the TGA result. The resulting dispersions were analyzed for the evaluation of dispersibility using an OM (BX43-P, OLYMPUS, Tokyo, Japan). Particle size distribution of the dispersion (Mastersizer 3000E, Malvern Panalytical, Malvern, UK) was conducted to confirm the degree of dispersion of CNTs. Finally, the adhesion of the dispersing agents to the CNT surface was verified through TEM (ELSZ-2000ZS, Otsuka, Hirakata, Japan).

### 2.4. Contact Angle Measurement

Contact angle measurements were conducted to evaluate the compatibility between the CNT dispersion and the cement prepared using the aforementioned method. The cement board was manufactured with a fixed water-to-cement (W/C) ratio of 0.3 using a planetary paddle mixer. A compact cement board, measuring 50 mm × 50 mm × 20 mm, was created by filling the mold with the blended cement. Every cement specimen underwent hydration for periods of 3, 14, and 28 days. A volume of 1.5 μL of both water and CNT dispersion was dropped onto the hydrated cement, and the contact angle was then measured. A larger angle means weaker interaction, while a smaller angle indicates better adhesion. This aids in evaluating cement nanocomposite properties. Contact angle measurements were conducted using the Phoenix 300 (SEO Co., Ltd., Suwon, Republic of Korea).

### 2.5. Fabrication and Characterization of Cement Nanocomposites

Cement pastes were formulated, each with varying ratios of dispersing agents in CNT dispersions. The cement nanocomposite was manufactured by maintaining a constant CNT concentration of 0.1 wt% of the cement and keeping the W/C ratio unchanged at 0.3. Based on the previous experimental results and literature, it has been confirmed that high concentrations of CNT (above 0.1 wt% relative to cement) do not contribute to the improvement of cement strength [13]. In this study, the CNT content was fixed at 0.1 wt%, and the research focus was on determining the optimal dispersing agent ratio. Using a planetary paddle mixer, the cement was thoroughly blended and then poured into a 50 mm × 50 mm × 50 mm mold. To prevent moisture evaporation from the sample, plastic wrap was employed to cover the top of the mold, and it was stored in the laboratory under ambient conditions for a day. After one day, the specimens were extracted from the molds and immersed in a saturated lime solution at 23 ± 2 °C for durations of 3, 14, and 28 days. Compressive strength assessments were carried out in accordance with the ASTM C 109 Standard Test Method for compressive strength of hydraulic cement mortars, utilizing 2-inch or 50-mm cube specimens. The compressive strength was assessed with a digital electric compressive strength tester (HS-1472, Hanshin Kumpoong. Co., Ltd., Gimpo, Republic of Korea).

### 2.6. Microstructure Analysis

After measuring the compressive strength, XRD analysis was carried out to examine the internal components of the nanocomposites. XRD analyses were executed using a Miniflex 300 Desktop X-Ray Diffractometer (Rigaku, Tokyo, Japan) with settings of 30 kV/15 mA and CuK radiation (λ = 1.5418 Å). In addition, FE-SEM was used to observe the morphology of MWCNTs within the nanocomposites. Microstructure examination was performed with the LEO SUPRA 55 equipment (Carl Zeiss, Oberkochen, Germany).

## 3. Results and Discussion

### 3.1. Determine the Mixing Ratio of Polymer Dispersing Agents to CNTs Using TGA Analysis

**Figure 2:**
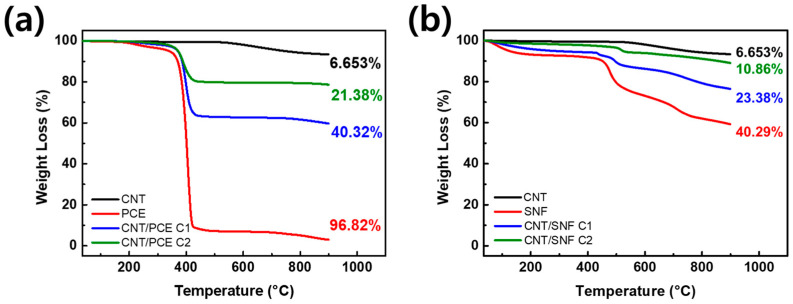
TGA graphs of (**a**) CNT/PCE and (**b**) CNT/SNF after the first (C1) and second centrifuge (C2) processes.

A centrifuge was utilized to separate the dispersing agent that had effective interaction with CNTs from the portion that had poor interaction within the CNT:SNF and CNT:PCE dispersion at a ratio of 1:2. During the centrifugation process, the dispersing agents that interacted with the CNTs settled and formed a precipitate, while those that did not adhere to the CNTs remained dissolved in the supernatant. The TGA results for the sediment formed during the centrifugation process enabled us to determine the percentage of polymer dispersing agent adhered to CNTs (Figure 2). CNTs experienced a weight loss of approximately 6.65% up to a temperature of 900 °C. In the case of PCE, roughly 96.82% of its weight was incinerated, while SNF underwent a loss of about 40.29%. The weight loss values of both CNTs and dispersants serve as the benchmark for determining the ratio, which was ascertainable by assessing the weight loss of the sediment after centrifugation. At the initial centrifuge step, CNT/PCE exhibited a weight loss of about 40.32%, followed by a subsequent weight reduction of 21.38% during the second centrifuge process. Additionally, for SNF, the initial procedure resulted in a 23.38% weight loss, with a subsequent 10.86% decrease in the second procedure. The specific ratio of polymer dispersing agents to CNT was determined by applying the provided equation, with the results obtained from TGA analysis.

$$\frac{X_{c}\%}{100}\times \left(100-Y_{d}\right)+\frac{X_{d}\%}{100}\times Y_{d}=\frac{X_{c/d}\%}{100}\times 100$$

$X_{c}$, CNT weight loss; $X_{d}$, dispersing agents weight loss; $X_{c/d}$, CNT/dispersing agents hybrid weight loss; $Y_{d}$, the ratio of dispersing agents ratio to CNT.

As a result, the amount of polymer dispersing agent in the sediment gradually diminishes with the number of centrifuge cycles. During the first centrifuge process, PCE remains at about 37% compared to CNT (CNT/PCE C1) and decreases to about 16% during the second process (CNT/PCE C2) (Figure 2a). For SNF, there is a tendency to decrease to 50% during the first centrifuge process (CNT/SNF C1) and further down to 13% in the second process (CNT/SNF C2) (Figure 2b). Due to the hydrophilic nature of both the backbone and side chain of PCE, their interaction with CNTs is limited [14]. On the other hand, the naphthalene-based structure of SNF allows for its integration with CNT, resulting in significant adhesion [15]. So even after centrifuge, a larger amount of SNF remains than PCE. These results suggest that dispersing agents with limited binding to CNTs can be gradually separated and eliminated as the centrifugation process advances. This is because the polymer dispersing agents lack interaction with the CNTs, dissolve in water, and do not accumulate in the sediment. Therefore, through the centrifuge process, the concentration of CNTs and polymer dispersing agents can be optimized.

### 3.2. OM Image Analysis

**Figure 3:**
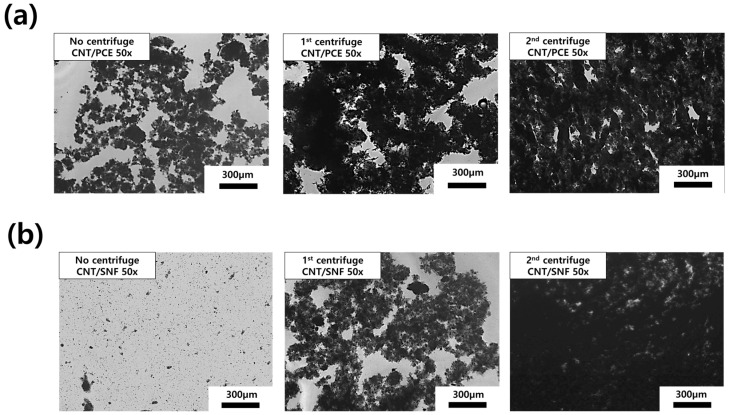
OM images of (**a**) CNT/PCE and (**b**) CNT/SNF dispersion.

Figure 3 shows OM images of the dispersion in which CNTs, initially precipitated using a centrifuge, are subsequently redispersed. In general, as the centrifugation process was repeated, the degree of dispersion of CNTs tends to gradually decrease. This is because the amount of polymer dispersing agent decreases throughout the centrifugation process. In the presence of PCE, some agglomeration of CNTs was observed after the first centrifugation and redispersion process (CNT/PCE C1) (Figure 3a). Furthermore, after the second process (CNT/PCE C2), the CNTs exhibited a shape indicating minimal dispersion. On the other hand, in the case of SNF, it is evident that the degree of CNT dispersion was superior to that of CNT/PCE (Figure 3b). Even after the first centrifugation and redispersion process, CNT/SNF C1 exhibited dispersibility comparable to CNT:PCE dispersion with no centrifugation (CNT:PCE 1:2) sample. However, when the centrifuge was run twice, it was observed that CNTs tended to aggregate in a manner similar to that seen with PCE (CNT/SNF C2). Because of its hydrophilic backbone and side chains, PCE exhibits lower dispersibility compared to SNF, which possesses a naphthalene structure [14,15]. In other words, the interaction between PCE and CNT is lower than SNF, leading to a larger amount of the polymer dispersing agent being removed through the centrifugation process. Therefore, upon redispersion, PCE exhibits a lower level of CNT dispersion compared to SNF.

### 3.3. Particle Size Distribution Analysis

**Figure 4:**
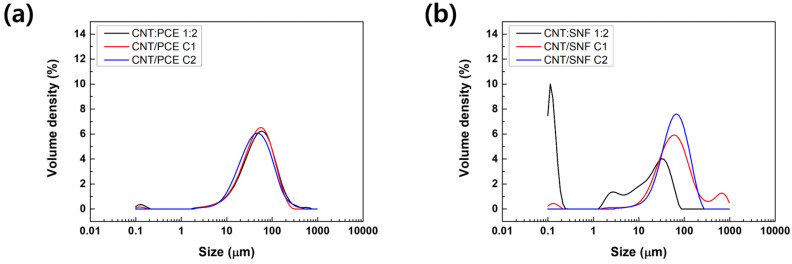
Particle size distribution graphs of (**a**) CNT/PCE and (**b**) CNT/SNF.

Particle size analysis was performed to assess both the particle size and the degree of dispersion of CNTs in the solution. Figure 4 and Table 1 show the results of particle size analysis. Upon examining Figure 4, CNT:SNF 1:2 and CNT:PCE 1:2 exhibit a notable presence of ultra-small particles (submicron size), which gradually diminished as the centrifugation process progressed. These small particles represent polymer-dispersing agents that do not interact with CNTs [16,17]. Through the repeated operation of the centrifuge, the portion of smaller particles disappeared, confirming the removal of any remaining residues. The results indicate that the remaining dispersing agents in CNT:SNF 1:2 and CNT:PCE 1:2 can be effectively separated and filtered through centrifugation.

With the exception of CNT:PCE C1, there is a tendency for the Dv[50] particle size to increase as the centrifugation process advances (Table 1). During the dispersion process, a discernible trend was observed, with the CNT particle size increasing and the degree of dispersion decreasing as the centrifuge progressed. In the particle size analysis for CNT:PCE dispersion, as the centrifuge process continued, there was a decrease in the specific surface area while the average area steadily increased. This indicates a gradual enlargement in the size of CNTs within the dispersion. However, when comparing the particle sizes using Dv[50], C1 dispersion exhibited a comparable value with that of the CNT:PCE 1:2 dispersion. On the other hand, when considering CNT:SNF dispersions, as a substantial excess of dispersing agent was removed, a notable decrease in the specific surface area of the particles was coupled with a significant increase in the average particle size. The findings indicate that the effectiveness of dispersion diminishes as the centrifugation process advances.

The effectiveness of the polymer dispersing agents significantly influenced the outcomes of particle size analysis. Specifically, when utilizing PCE, as observed through OM images (Figure 3), it exhibited limited efficacy in dispersing CNTs. Consequently, even though a substantial portion of the PCE adhered to the CNTs, a relatively high particle size distribution was recorded (Dv[50] of 53.6 µm for CNT:PCE 1:2 dispersion). The size distribution uniformity persisted even as excess PCE diminished during the centrifugation process. In contrast, when employing SNF, the dispersing ability of CNTs was notably superior, leading to a wider range of CNT particle sizes (Figure 4b CNT:SNF 1:2). Subsequently, particle size distribution rapidly increased based on the reduction in excess SNF through the centrifugation process.

### 3.4. FE-TEM Analysis of CNT Dispersions

**Figure 5:**
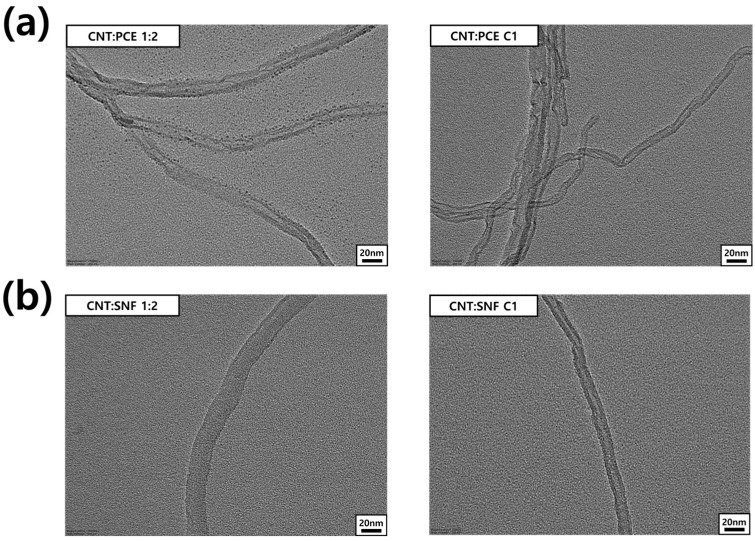
FE-TEM images of CNTs in (**a**) CNT/PCE and (**b**) CNT/SNF dispersions.

Figure 5 reveals the morphology of CNTs within the dispersing solution. FE-TEM images were utilized to compare the change in thickness of CNTs before and after centrifugation. The pristine CNTs have a thickness within the range of 10–13 nm. At a CNT:PCE ratio of 1:2, CNTs demonstrate a thickness ranging from 18 to 21 nm (Figure 5a and Figure S1 in the supplementary data). The excessive PCE dispersing agent adhering to the CNT surface led to a thicker diameter compared to the pristine CNTs. Moreover, PCE that has not adhered to the CNTs was present in the vicinity, forming a dot-like morphology [18]. After the centrifugation process, any excess PCE that has not reacted with the CNTs disappeared, resulting in a thickness that closely resembles that of the pristine CNTs (Figure 5a).

A distinctly thicker diameter of CNT (over 20 nm) can be observed in the CNT:SNF 1:2 dispersion compared to the pristine CNTs (Figure 5b). The increased thickness can be attributed to the surplus SNF adhering to the CNT, primarily due to π–π bond interactions [15]. However, after the centrifugation step, there is a decrease in the SNF content, leading to a restoration of a CNT thickness (10–13 nm). In other words, the FE-TEM image enables verification of the excess dispersant attached around the CNT. Also, it can be confirmed that, through the centrifuge process, only the minimum dispersing agent for CNT dispersion remained on the CNT surface, resulting in a thickness similar to that of pristine CNTs (Figure S2 in the supplementary data). Results from OM and particle size analysis confirm that this minimal amount of dispersing agent represents the optimal ratio for effectively dispersing CNTs.

### 3.5. Contact Angle Analysis

Since CNTs exhibit poor interfacial compatibility with cement [10], it is necessary to apply cement superplasticizers to enhance the compatibility at the interface between the CNT dispersion and cement. To assess the influence of PCE and SNF on the interfacial affinity between CNT and cement, a contact angle analysis was conducted.

**Figure 6:**
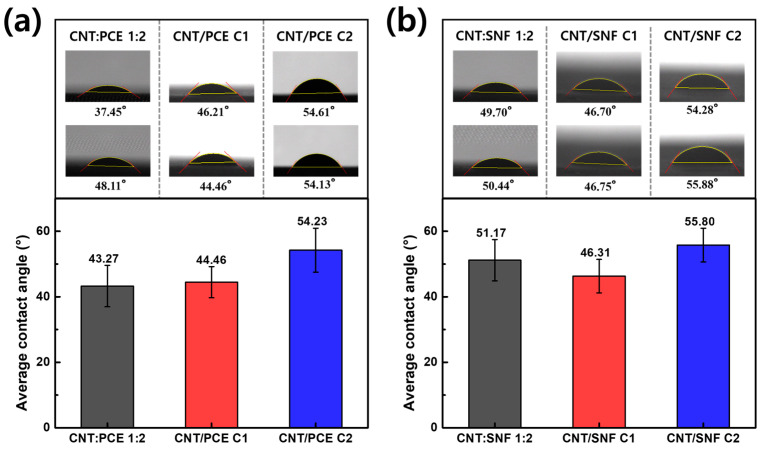
Contact angle between CNT dispersion and plain cement substrate with (**a**) PCE and (**b**) SNF dispersing agents.

Figure 6 exhibits the changes in contact angle between the CNT dispersions and cement substrate at different concentrations of polymer dispersing agent throughout the centrifugation process. In Figure 6a, a gradual increase in the contact angle between the CNT dispersion and cement can be observed as the centrifugation process advances. In the CNT:PCE C2 sample, the contact angle is recorded at 54.24°, representing a considerable increase compared to the measurement obtained in CNT:PCE 1:2 (43.27°) and C1 (44.46°). Since the PCE dispersing agent has a COO^−^ functional group on its surface, it can interact with the Ca^2+^ of cement, facilitating a closer connection between cement and CNT dispersion [19]. The compatibility between the dispersion and cement, along with the quantity of PCE, exhibited a rapid decrease as the centrifuge continued. In Figure 4, the particle size analysis of CNT/PCE revealed that the amount of the residual dispersing agents, not interacting with the CNT surface, was relatively low, which resulted in minimal change in the contact angle of the CNT:PCE C1 with CNT:PCE 1:2. After the second centrifugation, the rapid increase in the contact angle suggested the removal of the dispersing agents that were initially interacted with the CNTs.

The interaction between SNF and cement through electrostatic forces, coupled with its capacity to induce water-reducing reactions, led to a larger contact angle compared to the use of PCE [20] (Figure 6b). Furthermore, in contrast to PCE, SNF exhibited the lowest contact angle following the first centrifugation process. This outcome implies that the compatibility with cement is improved when only the SNF interacting with CNTs is present, rather than an excess of SNF. In contrast, in the CNT/SNF C2 sample, the contact angle tended to increase rapidly due to a reduction in the amount of interacted SNF. In summary, in both cases using PCE and SNF, the C1 samples, which exclusively included dispersing agents interacting with CNTs, demonstrated the highest compatibility with cement.

### 3.6. Compressive Strength Test

**Figure 7:**
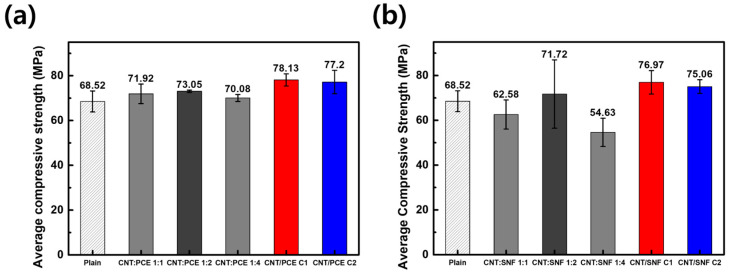
Compressive strength results of cement nanocomposites according to mixing ratio of CNT to dispersing agent (**a**) PCE and (**b**) SNF.

Figure 7 displays a graph comparing the overall compressive strengths between the previous and the current experiments [21]. In the last experiment, the cement nanocomposite with a CNT to PCE ratio of 1:2 exhibited the highest compressive strength at 73.05 MPa, representing an approximately 6.6% increase compared to plain cement. The cement nanocomposite with a CNT:SNF 1:2 displayed a lower strength compared to CNT:PCE 1:2-containing cement but still achieved a higher value (71.72 MPa) than those of other CNT:SNF samples. However, the cement nanocomposites derived from the initial centrifuge treatment demonstrated higher compressive strengths compared to those produced with a fixed ratio. In the case of the cement nanocomposite with CNT/PCE C1, the compressive strength measured 78.13 MPa, representing an approximately 14% improvement over plain cement. Meanwhile, in CNT/SNF C1, it reached 76.97 MPa, showcasing about a 12.3% enhancement.

In composites prepared with a fixed ratio (1:1, 1:2, and 1:4), apart from the polymer dispersing agents that adhere to the CNT, the residual quantity was suspended within the cement composites. The residual dispersing agent within the mixture slows down cement hydration, while concurrently, the CNTs inserted between cement pores promote hydration and enhance strength [6,11]. When using the C2 samples, the composites exhibited weaker strength than C1. This phenomenon can be attributed to the poorer dispersibility of CNT dispersions and their compatibility with cement compared to the C1 samples. In other words, the initial centrifugation step optimizes the concentration of the polymer dispersing agent for the CNT dispersion and enhances its compatibility with the cement, leading to the utmost mechanical properties for the composite materials.

### 3.7. XRD Analysis of Cement Composites

**Figure 8:**
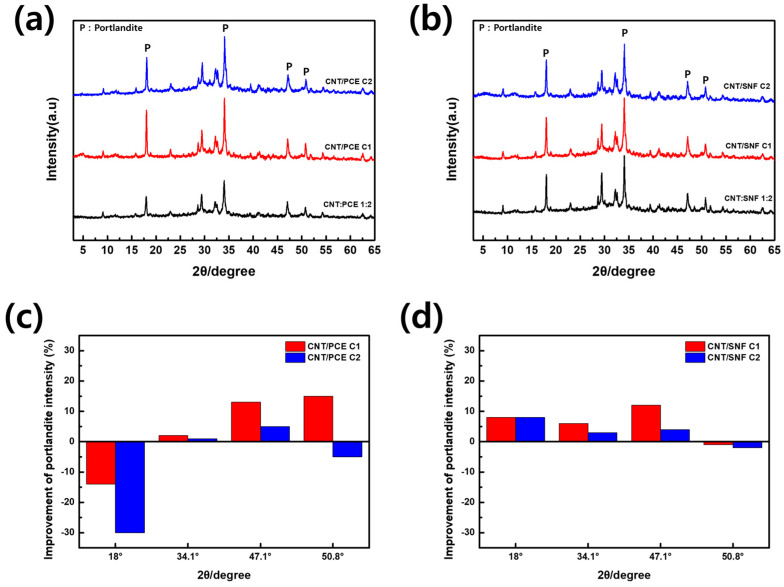
XRD analysis of cement nanocomposites with (**a**) CNT/PCE and (**b**) CNT/SNF and enhancement of C-S-H and portlandite peaks of cement nanocomposites with (**c**) CNT/PCE and (**d**) CNT/SNF C1 and C2 dispersion in comparison to those of the CNT:PCE 1:2 and CNT:SNF 1:2.

Figure 8a,b depicts XRD analysis graphs of cement composites made up of centrifuge-treated and untreated CNT dispersions. In the XRD pattern of cement composites, distinct peaks corresponding to calcium hydroxide (portlandite, marked as ‘P’ on the graph) are observable. These peaks are the outcome of the hydration process [16,22,23]. The intensity of the portlandite peaks, which can be considered an indirect indicator of calcium silicate hydration, exhibited variations based on the type of dispersing agent and the number of centrifugation cycles.

Figure 8c,d specifically depicts the XRD peaks attributed to cement hydration, allowing for a detailed quantitative comparison of the intensity of each peak. The graphs provide a comparative analysis of the enhanced intensity of the portlandite peak in comparison to those of the cement nanocomposites with CNT:PCE 1:2 and CNT:SNF 1:2, respectively. Upon observing Figure 8c, both CNT/PCE C1 and C2 nanocomposites display higher portlandite peak intensities than CNT:PCE 1:2, except for the 18° Portlandite peak. In particular, cement nanocomposite with CNT:PCE C1 dispersion had the highest intensity, up to 15% enhancement compared to CNT:PCE 1:2 nanocomposite. Similarly, in Figure 8d, all peaks, except for the 50.8° peak, exhibited higher intensities than CNT:SNF 1:2 nanocomposite, suggesting a more pronounced presence of portlandite in both CNT/SNF C1 composite. The dispersing agents are recognized for slowing down the hydration rate of cement. However, applying the CNT dispersion with the optimized dispersing agent concentration to the cement composite through centrifugation results in the most rapid hydration rate, ultimately achieving enhanced compressive strength.

### 3.8. FE-SEM Analysis of Cement Nanocomposites

**Figure 9:**
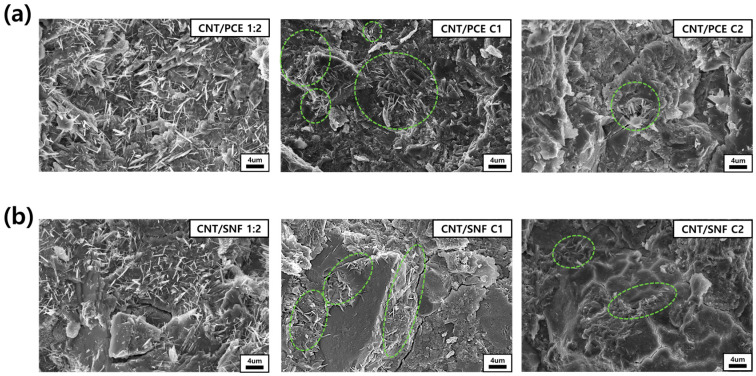
FE-SEM images of cement nanocomposites of (**a**) CNT/PCE and (**b**) CNT/SNF.

Figure 9 displays FE-SEM images illustrating the internal structure of the cement composites, where the degree of distribution of CNTs inside the cement was compared according to centrifugation treatment. The FE-SEM images of the cement nanocomposites with CNT/PCE 1:2 and CNT:SNF 1:2 dispersion reveal a homogeneous distribution of carbon nanotubes within the cement matrix. Specifically, the uniform distribution of CNTs was most pronounced when utilizing PCE as the dispersing agent. However, as the centrifugation process advances, there is a gradual decrease in the uniformity of CNT distribution. After a single round of centrifugation, it becomes apparent that CNTs tend to aggregate sporadically. Furthermore, when the centrifugation procedure was performed twice, CNT bundles tended to aggregate in specific areas, attributed to their limited dispersion capabilities, as observed in the OM images (Figure 3). However, considering that the majority of identified CNTs were located in close proximity to cracks within the cement structure, their presence may positively contribute to the enhancement of the strength of cement composites.

Through a comparison of OM images of CNT dispersion with FE-SEM images of cement nanocomposites, it is evident that the distribution of CNTs within the cement was influenced by the degree of CNT dispersion. Previous studies have highlighted that achieving a proper dispersion of CNTs is crucial for their uniform distribution within the cement matrix, which in turn contributes to enhancing the strength of the cement. However, compared to the compressive strength results obtained in this study, distributing the CNTs so that they tightly hold the pores inside the cement (cement nanocomposite from C1 and C2 dispersions) was more effective in enhancing the compressive strength than spreading the CNTs evenly throughout [24]. Indeed, high-magnification FE-SEM images confirmed that CNTs were concentrated around the cement pores after the centrifugation process (Figure S3 in the supplementary data). Furthermore, upon closer examination of the interior of the cement crack, it was observed that CNTs became embedded between the cement matrix, forming robust CNT-cement interface bonding (Figure S4 in the supplementary data). As indicated by the FE-SEM examination, the process of optimizing the concentration of CNTs and dispersing agents during centrifugation proves to be a more effective method for preventing the development of cracks within the cement.

## 4. Conclusions

This study aims to improve the strength of cement composites through the optimization of the ratio between CNTs and dispersing agents. The residual dispersing agents, which did not adhere to the CNTs, were eliminated via centrifugation, and the ratio was validated using TGA. As a result, following the initial centrifugation, approximately 37% of PCE and 50% of SNF remained in comparison to CNTs. After conducting a comparative analysis of OM images and particle size analysis, the dispersing agents at the optimized ratio efficiently facilitated the dispersion of CNTs. Furthermore, it was verified that the contact angle remained largely unchanged even after centrifugation, indicating that there were no issues with the interfacial compatibility with cement. Consequently, the compressive strength of the cement composites was improved, reaching 78.13 MPa for CNT/PCE C1 and 76.97 MPa for CNT/SNF C1. These improved strengths are 14.0% and 12.3%, respectively, compared to plain cement (68.52 MPa). Microstructural analysis reveals that CNTs serve a dual role within cement composites. First, XRD analysis demonstrates that CNTs facilitated the hydration process of cement, leading to the increased production of portlandite. This serves as evidence that an optimal combination of CNTs and dispersing agent could positively impact cement, enhancing its properties as the hydration level increased. Second, CNTs can play a bridge role in stabilizing pores in cement. Connecting pores served to decelerate the rate of crack formation, providing an additional reinforcement effect on the cement nanocomposites. FE-SEM analysis reveals that the CNTs with the optimized amount of dispersing agents could enhance strength by effectively filling and bridging cracks in the cement matrix. The cement nanocomposites derived from the optimization procedure involving dispersion and centrifugation exhibit potential applicability for enhancing safety standards within the construction industry. Furthermore, for the development of special-purpose construction materials that require greater strength, such as those used in high-rise buildings or construction for extreme environments, the use of cement nanocomposites reinforced with CNTs can serve as a practical solution.

## Figures and Tables

**Table 1:** The results of particle size distribution analysis.

	Specific Surface Area (m^2^/kg)	D [3,2] (µm)	Dv[50] (µm)
CNT:PCE 1:2	823.6	7.28	53.6
CNT/PCE C1	455.0	13.2	51.3
CNT/PCE C2	193.5	31.0	70.5
CNT:SNF 1:2	17,620	0.341	6.28
CNT/SNF C1	969.9	6.19	63.0
CNT/SNF C2	126.7	47.4	65.9

D [3,2], average area of particles; Dv[50], corresponding particle size when the cumulative percentage reaches 50%.

## Data Availability

Data are contained within the article.

## Supplementary Materials

The following supporting information can be downloaded at: https://www.mdpi.com/article/10.3390/polym16030428/s1, Figure S1. (a) FE-TEM images of CNT/PCE dispersion and (b) histogram before and after the centrifugation. Figure S2. (a) FE-TEM images of CNT/SNF dispersion and (b) histogram before and after the centrifugation. Figure S3. High-magnification FE-SEM images of cement nanocomposites of (a) CNT/PCE and (b) CNT/SNF after centrifugation. Figure S4. High-magnification FE-SEM image of the interior of the crack in the cement nanocomposites using CNT/PCE C1.
