# Supplementary material for: Reinforcement of Cement Nanocomposites through Optimization of Mixing Ratio between Carbon Nanotube and Polymer Dispersing Agent
Source: Polymers (Basel). 2024 Feb 3;16(3):428. doi: 10.3390/polym16030428 (PMC10857042; doi:10.3390/polym16030428)
Supplement: Supplementary file 1 [file polymers-16-00428-s001.zip › polymers-2841569-supplementary.pdf]

Supplementary data

Reinforcement of cement nanocomposites through optimization of mixing ratio between carbon nanotube and polymer dispersing agent

Seok Hwan An, Ki Yun Kim, and Jea Uk Lee*

[a] *Department of Advanced Materials Engineering for Information and Electronics, Integrated Education Institute for Frontier Science and Technology (BK21 Four), Kyung Hee University, Gyeonggi-do, 17104, Republic of Korea*

* Corresponding author: Jea Uk Lee; E-mail: leeju@khu.ac.kr

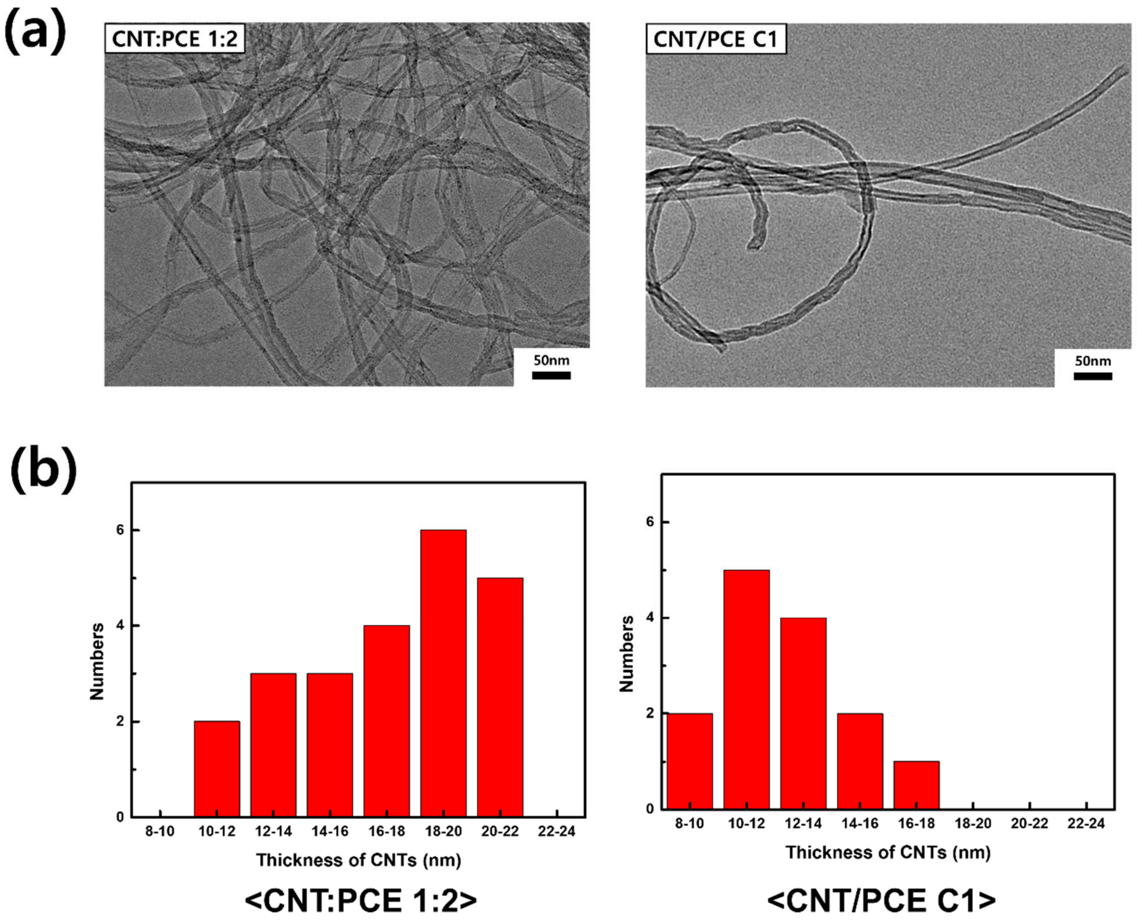

Figure S1. (a) FE-TEM images of CNT/PCE dispersion and (b) histogram before and after the centrifugation.

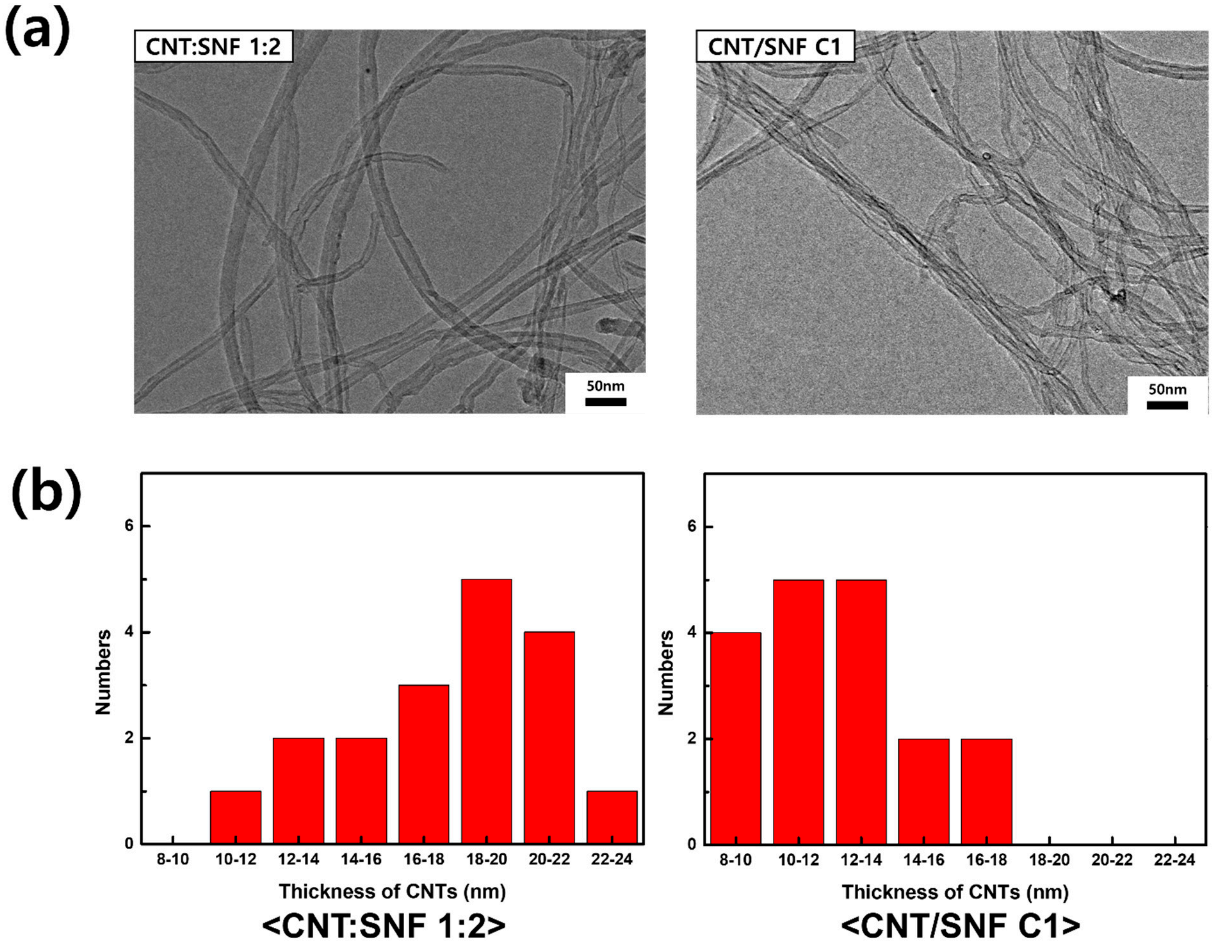

Figure S2. (a) FE-TEM images of CNT/SNF dispersion and (b) histogram before and after the centrifugation.

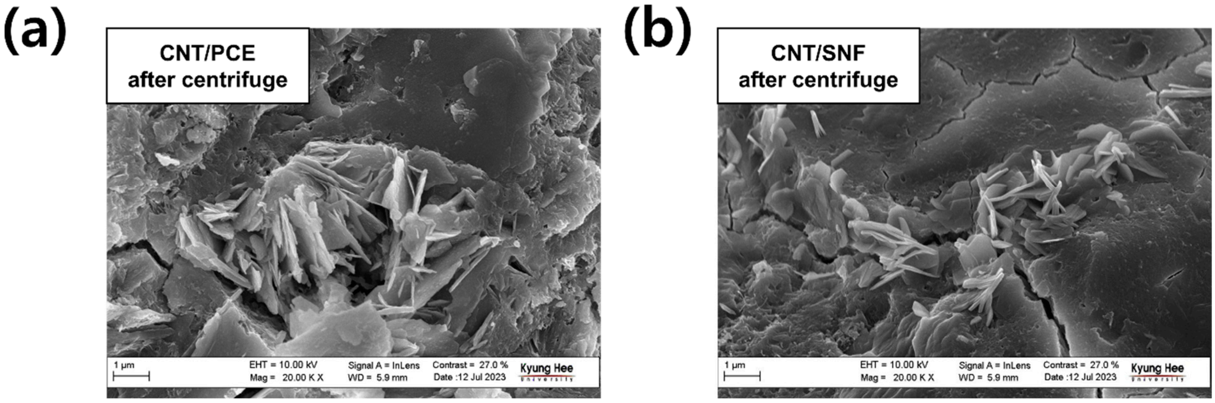

Figure S3. High-magnification FE-SEM images of cement nanocomposites of (a) CNT/PCE and (b) CNT/SNF after centrifugation.

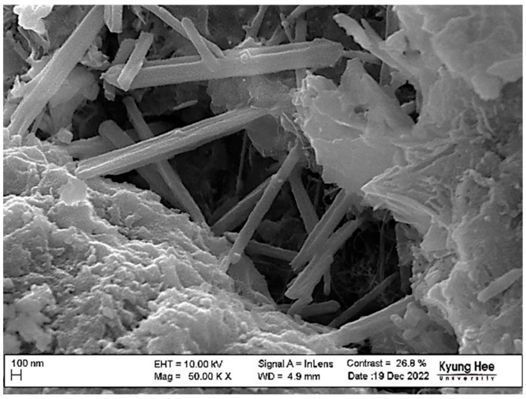

Figure S4. High-magnification FE-SEM image of the interior of the crack in the cement nanocomposites using CNT/PCE C1.
